# Clinical significance and predictors of oncologic outcome after radical prostatectomy for invisible prostate cancer on multiparametric MRI

**DOI:** 10.1186/s12885-018-4955-8

**Published:** 2018-11-01

**Authors:** Doo Yong Chung, Dong Hoon Koh, Hyeok Jun Goh, Min Seok Kim, Jong Soo Lee, Won Sik Jang, Young Deuk Choi

**Affiliations:** Department of Urology, Urological Science Institute, Yonsei University College of Medicine, 50-1, Yonsei-ro, Seodaemun-gu, Seoul, 120-752 Korea

**Keywords:** Prostatic neoplasms, Magnetic resonance imaging, Prostatectomy, Prognosis

## Abstract

**Background:**

The objective of our study was to evaluate the clinical significance of invisible prostate cancer (iPCa) on multiparametric magnetic resonance imaging (mpMRI) by analyzing clinical parameters and oncologic outcomes.

**Methods:**

We retrospectively reviewed the records of patients treated with radical prostatectomy (RP) from 2010 to 2015 at our institution. Before RP, all patients were confirmed to have prostate cancer based on prostate biopsy. We excluded patients who underwent neoadjuvant therapy. Additionally, we excluded patients who had incomplete mpMRI based on PI-RADS (Prostate Imaging Reporting and Data System). iPCa was defined as having no grade 3 or higher region of interests using a scoring system established by PI-RADS without limitations on interpretation from mpMRI by radiologists. We selected patients with iPCa using this protocol. We analyzed data using univariate and multivariate cox regression analysis, logistic analysis, Kaplan-Meier curves, and receiver operator characteristic curves to predict biochemical recurrence (BCR).

**Results:**

A total of 213 patients with iPCa were selected according to the patient selection protocol. Among them, pathological findings showed that Gleason score (GS) G6, G7 and ≥ G8 were present in 115 cases (54.0%), 78 cases (36.6%), and 20 cases (9.4%), respectively. Further, extracapsular extension (ECE), positive surgical margins (PSM), and lymphovascular invasion (LVI) were present in 28 (13.1%), 18 (8.5%), and 3 cases (1.4%), respectively. Seminal vesicle invasion (SVI) was observed in one case (0.5%). During a median follow-up time of 51 months, BCR was observed 29 cases. Adverse pathology (AP) was defined as GS ≥8, ECE, SVI and LVI. AP and prostate specific antigen (PSA) were significantly associated with BCR. Moreover, PSA > 6.2 ng/ml was suggested as a cut-off value for predicting BCR.

**Conclusions:**

In our results, cases of iPCa had clinically significant PCa, and AP and poor prognosis were also observed in some. Additionally, we found that PSA is the most clinically reliable predictor of oncologic outcome. We suggest that active treatment and diagnosis should be considered for patients with iPCa with PSA > 6.2 ng/ml.

## Background

Prostate cancer (PCa) is the most common type of newly diagnosed malignancy in males [1], and it accounts for nearly 30% of all diagnosed male cancers [2]. In 2012, around 1,111,700 new cases and 307,500 deaths were recorded worldwide [3]. As PCa screening by measuring prostate-specific antigen (PSA) levels has become more widespread, the proportion of PCa presenting with low-risk factors has also increased. [4] According to the European Association of Urology guidelines, an extended 12-core systematic transrectal ultrasound (TRUS)-guided biopsy should be performed for patients with an elevated prostate specific antigen (PSA) level, which is endorsed as the optimal biopsy method [5]. However, this diagnostic strategy has disadvantages based on random sampling and is largely operator dependent. [6]

Accordingly, several complementary measures are being developed. Since its first usage in 1983, magnetic resonance imaging (MRI) has increasingly been used for PCa diagnosis because of its growing availability, multiparametric imaging, and the combination of anatomic and functional data [7]. The accuracy of multiparametric magnetic resonance imaging (mpMRI) has not only been established for biopsy specimens, but also for histopathologic correlations using prostatectomy specimens. Recent publications have demonstrated detection rates of significant PCa between 80 and 96% for MRI compared to whole-mount sections [8–10] Therefore, imaging techniques have played an increasingly important role in the management of localized PCa. Moreover, mpMRI-TRUS fusion-targeted biopsy represents a substantial step forward in the detection of PCa. [11] Therefore, the clinical significance of invisible PCa on mpMRI tends to be overlooked.

However, despite advances in imaging and target biopsy, mpMRI still produces some false negatives and false positives [12–14], and not all PCa is detected by mpMRI. Therefore, the aim of our study was to evaluate the clinical significance of invisible prostate cancer (iPCa) on mpMRI. We examined clinical and pathological characteristics to evaluate oncology outcomes following radical prostatectomy (RP) in patients with iPCa.

## Methods

### Study design and patients

We retrospectively retrieved the clinical and pathological data of 3057 individuals with PCa who underwent mpMRI before RP at our institution between January 2010 and December 2015. Among them, patients who had undergone androgen deprivation therapy (ADT), or radiation therapy (RT) before the RP were excluded from the study. Before RP, all patients were diagnosed with PCa through a TRUS-guided 12-core systematic needle biopsy.

Additionally, we reviewed only patients with mpMRI based on the Prostate Imaging Reporting and Data System (PI-RADS) [15, 16], including standardized criteria for Likert scoring of multiparametric sequences (T1-weighted [T1W] and T2-weighted [T2W] imaging, diffusion-weighted imaging [DWI], apparent-diffusion coefficient [ADC] and dynamic contrast enhanced imaging [DCE]) using a 3.0 T MRI system (Intera Achieva 3.0 T, Phillips Medical System, Best, The Netherlands). All images were retrospectively reviewed by three experienced uroradiologists who were blinded to biopsy results and who conducted a consensus review of the mpMRI images of all patients. In mpMRI, suspicious lesions were graded 1–5 using a scoring system established by PI-RADS. Negative MRI findings were defined as having no grade 3 or higher region of interests without limitations on interpretation from mpMRI by radiologists. We defined this as an iPCa [8, 12].

**Fig. 1:**
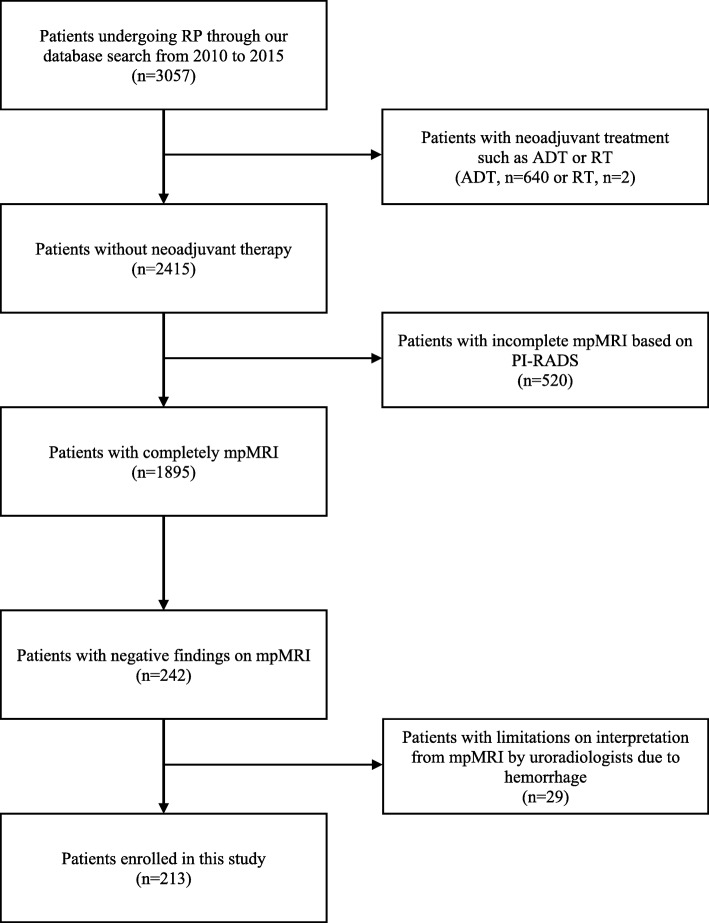
Flow chart of patient selection

Of 3057 patients, 642 (640 ADT, 2 RT) (642/3057, 21.0%) had neoadjuvant therapy. Of the remaining 2415 patients, 520 (520/2415, 21.53%) underwent incomplete mpMRI based on PI-RADS. The remaining 1895 patients underwent image review by radiologists, of whom 242 patients (242/1895, 12.8%) had negative findings on mpMRI. Of these, 29 patients who had limited image interpretation due to hemorrhage were excluded. As a result, a total of 213 patients who had an invisible tumor on mpMRI of clinical stage T1c were enrolled in our study. (Fig. 1).

Clinical characteristics of these patients including age, body mass index (BMI), accompanying medical history (hypertension, diabetes mellitus), preoperative PSA, prostate volume measured by TRUS, Gleason score (GS) following prostate biopsy, and pathologic characteristics of specimens following RP were obtained through a review of medical records at our institution. All pathologic diagnosis was performed by expert pathologists. Biopsy specimens obtained from outside of our hospital were reviewed by our pathologists. Moreover, the pathologists reviewed the deficiencies of the pathological report of 213 patients included in our study and confirmed that there was no problem. Adverse pathology (AP) was defined as GS ≥8, extracapsular extension (ECE), seminal vesicle invasion (SVI), and lymphovascular invasion (LVI) [17–19].

Finally, the TNM stage was determined according to the 8th edition of the American Joint Committee on Cancer TNM staging system.

### Follow-up

Postoperative PSA follow-up was performed monthly for the first 6 months, every 3 months for the second year, and every 6 months thereafter. Biochemical recurrence (BCR) was defined as any two consecutive increases in serum PSA ≥0.2 ng/ml following RP [20]. BCR-free survival was defined as the time from RP to BCR. The follow-up period was calculated from the time of RP to the date of the last known contact with the patient.

### Statistical analysis

Univariate and multivariate Cox proportional hazards regression analyses were performed to assess the association between baseline parameters and BCR-free survival. In addition, univariate and multivariate logistic regression were carried out for the significant factors of AP, and the Kaplan-Meier method with log-rank tests were performed to estimate and compare oncologic outcomes according to AP and PSA.

Receiver operator characteristic (ROC) curve analysis was performed to determine optimal cut-off value via the area under the curve (AUC). A total AUC score of 0.8–0.9 was interpreted as an excellent level and 0.7–0.8 as a good level.

Significant variables from univariate analysis were included in the multivariate analysis. Comparisons with *p* < 0.05 were considered statistically significant. All statistical analyses were performed using SPSS Statistics software, version 23.0 (IBM, Armonk, NY, USA).

## Results

### Patient and disease characteristics

A total of 213 patients with iPCa were included, and their baseline clinical and pathological features are shown in Table 1.

**Table 1:** Baseline patient characteristics

	Median	IQR
Age, year	64	59–69
BMI, kg/m2	24.5	22.4–26.1
Comorbidity	N	%
Hypertension	92	43.2
DM	27	12.7
PSA level, ng/ml	5.35	4.0–7.5
Prostate volume, ml	31.2	23.2–41.2
Follow up period after RP, months	51	32–70
Tumor volume at specimen, cc	0.6	0.2–1.4
Biopsy Gleason score	N	%
6	162	76.1
7	37	17.4
≥ 8	14	6.6
Pathologic Gleason score	N	%
6	115	54
7	78	36.6
≥ 8	20	9.4
Pathologic T stage	N	%
≤ T2	185	86.9
≥ T3	28	13.1
ECE	N	%
	28	13.1
SVI	N	%
	1	0.5
PSM(R1)	N	%
	18	8.5
LVI	N	%
	3	1.4
PNI	N	%
	63	29.6
BCR	N	%
	29	13.6

*IQR* Interquartile range, *BMI* Body mass index, *DM* Diabetes mellitus, *PSA* Prostate-specific antigen, *RP* Radical prostatectomy, *ECE* Extracapsular extension, *SVI* Seminal vesicle invasion, *PSM* Positive surgical margins, *LVI* Lymphovascular invasion, *PNI* Perineural invasion, *BCR* Biochemical recurrence

The median age of patients was 64 years (interquartile range [IQR] 59–69). The median prostate volume, as measured by TRUS, was 31.2 ml (IQR 23.2–41.2), and the median PSA level was 5.35 ng/ml (IQR 4.0–7.5). The median follow-up from RP was 51 months (IQR 32–70). Among the patients, 92 had hypertension (43.2%) and 27 had diabetes mellitus (12.7%). Among the biopsy specimens, 162 had GS 6 (76.1%), 37 had GS 7 (17.4%), and 14 had GS ≥8 (6.6%).

In specimens following RP, 115 cases were GS 6 (54.0%) and 78 were GS 7 (36.6%). Furthermore, GS above 8 was reported in 20 cases (9.4%). Pathologic ≤T2 and ≥ T3 were reported 185 cases (86.9%) and 28 cases (13.1%), respectively. The median tumor volume of specimens following RP was 0.6 ml (IQR 0.2–1.4). ECE was present in 28 cases (13.1%), and surgical margins were involved in 18 cases (8.5%). SVI was observed in one case (0.5%). Perineural invasion was reported in 63 cases (29.6%) and LVI was present in 3 cases (1.4%). Lymph node metastasis was not reported. Furthermore, there were no cancer-specific deaths during the observation period.

### Oncologic outcomes and prognostic factors

During the follow up period, we observed 29 cases (13.6%) with BCR in this study. Univariate and multivariate Cox regression analyses were performed with each clinical parameter for BCR. In these analyses, preoperative PSA (hazard ratio [HR] 1.164, *p* < 0.001), GS ≥8 (HR 5.009, *p* = 0.004), pathologic T stage (HR 3.621, *p* = 0.003), and LVI (HR 5.129, *p* = 0.039) were all independent prognostic factors for BCR. (Table 2).

**Table 2:** Univariate and multivariate analyses of factors associated with biochemical recurrence

Variable	Univariate		Multivariate	
	HR (95% CI)	*p* value	HR (95% CI)	*p* value
Age, year	1.012 (0.960–1.067)	0.656		
PSA, ng/ml	1.198 (1.129–1.272)	< 0.001	1.164 (1.069–1.268)	< 0.001
BMI, kg/m2	0.943 (0.813–1.093)	0.433		
Prostate volume, ml	1.001 (0.981–1.023)	0.892		
Hypertension				
No	1 (Ref)			
Yes	1.548 (0.747–3.210)	0.240		
DM				
No	1 (Ref)			
Yes	0.849 (0.257–2.808)	0.789		
Biopsy Gleason score				
6	1 (Ref)		1 (Ref)	
7	1.952 (0.783–4.866)	0.151	0.957 (0.315–2.913)	0.939
≥ 8	7.052 (2.956–16.828)	< 0.001	0.985 (0.179–5.434)	0.986
Pathologic Gleason score				
6	1 (Ref)		1 (Ref)	
7	2.619 (0.982–6.989)	0.054	1.905 (0.653–5.560)	0.238
≥ 8	13.213 (4.879–35.778)	< 0.001	5.009 (1.664–15.076)	0.004
Tumor volume at specimen, cc	1.745 (1.263–2.411)	0.001	0.780 (0.499–1.218)	0.275
Pathologic T stage				
≤ T2	1 (Ref)		1 (Ref)	
≥ T3	9.721 (4.630–20.411)	< 0.001	3.621 (1.535–8.540)	0.003
PSM(R1)				
No	1 (Ref)		1 (Ref)	
Yes	3.935 (1.778–8.706)	0.001	0.800 (0.271–2.363)	0.686
LVI				
No	1 (Ref)		1 (Ref)	
Yes	8.455 (1.978–36.133)	0.004	5.129 (1.087–24.187)	0.039
PNI				
No	1 (Ref)			
Yes	1.677 (0.800–3.514)	0.171		

*HR* Hazard ratio, *PSA* Prostate-specific antigen, *BMI* Body mass index, *DM* Diabetes mellitus, *PSM* Positive surgical margins, *LVI* Lymphovascular invasion, *PNI* Perineural invasion

**Fig. 2:**
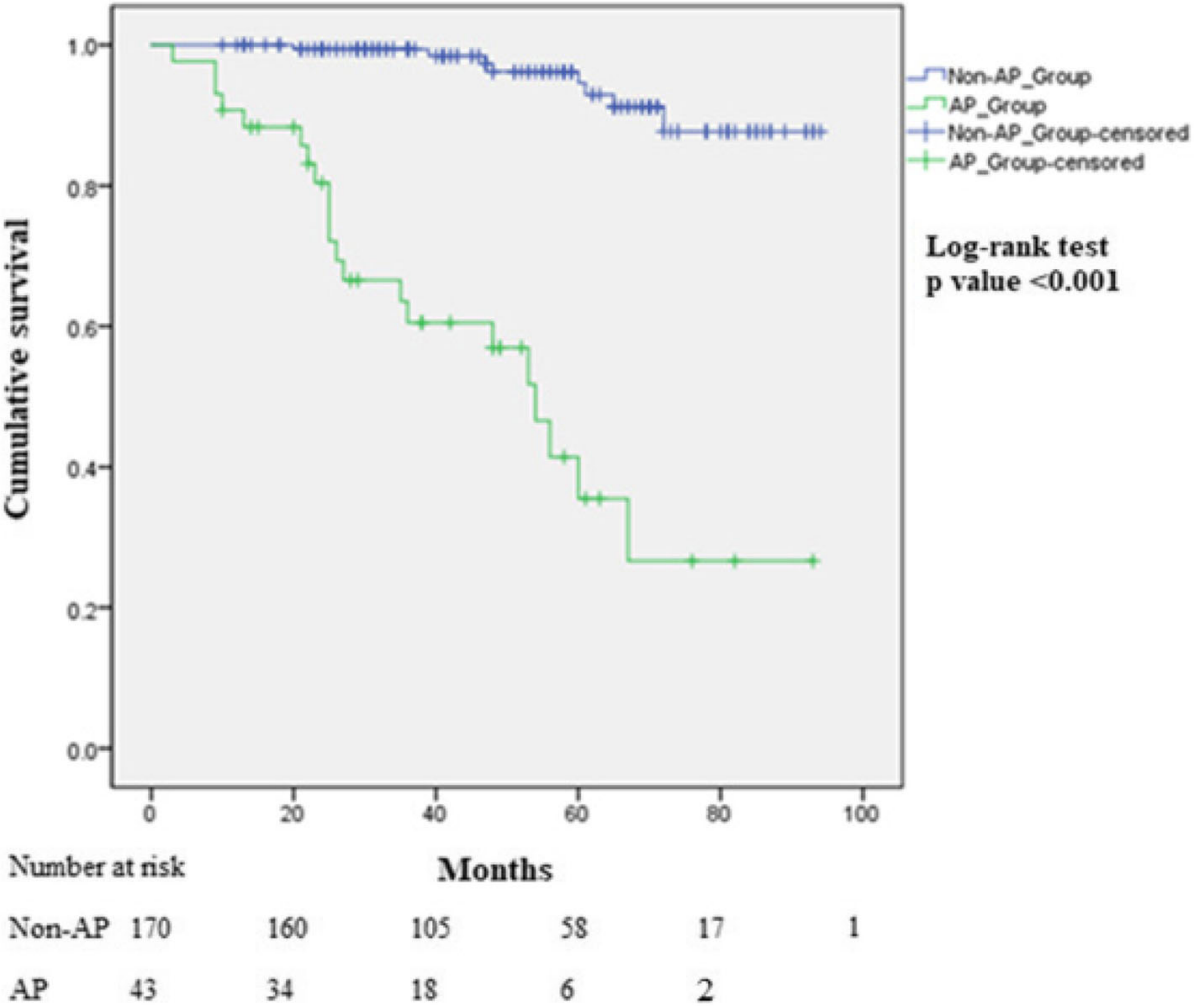
Kaplan-Meier curves for biochemical recurrence (BCR)-free survival in patients according to adverse pathology (GS ≥ 8, LVI, ECE and SVI)

AP (GS ≥ 8, LVI, ECE and SVI) and PSA were related to oncologic outcomes. In addition, 43 cases (20.2%) had statistically significant AP. We analyzed preoperative clinical parameters to predict AP associated with oncologic prognosis using univariate and multivariate logistic regression analyses, and found that preoperative PSA (odds ratio [OR] 1.270, *p* < 0.001), biopsy GS (GS 7 vs. GS 6: OR 4.353, *p* = 0.001; and GS ≥8 vs. GS 6: OR 28.076, p < 0.001) were independent prognostic factors for AP (Table 3). Additionally, Kaplan-Meier curves showed that BCR-free survival was significantly decreased in the AP group (log-rank test, p < 0.001). (Fig. 2).

**Table 3:** Univariate and multivariate analyses of factors associated with adverse pathology

Variable	Univariate		Multivariate	
	OR (95% CI)	*p* value	OR (95% CI)	*p* value
Age, year	1.032(0.982–1.083)	0.214		
PSA, ng/ml	1.291 (1.164–1.430)	< 0.001	1.270 (1.141–1.421)	< 0.001
BMI, kg/m2	0.972 (0.857–1.103)	0.660		
Prostate volume, ml	0.990 (0.965–1.015)	0.418		
Hypertension				
No	1 (Ref)			
Yes	1.683 (0.859–3.299)	0.129		
DM				
No	1 (Ref)			
Yes	1.151 (0.434–3.054)	0.778		
Biopsy Gleason score				
6	1 (Ref)		1 (Ref)	
7	4.870 (2.133–11.118)	< 0.001	4.353 (1.770–10.704)	0.001
≥ 8	29.333 (7.473–115.138)	< 0.001	28.076 (6.720–117.296)	< 0.001

*OR* Odds ratio, *CI* Confidence interval, *PSA* Prostate-specific antigen, *BMI* Body mass index, *DM* Diabetes mellitus

### ROC curve and cut-off value of PSA in relation to BCR

**Fig. 3:**
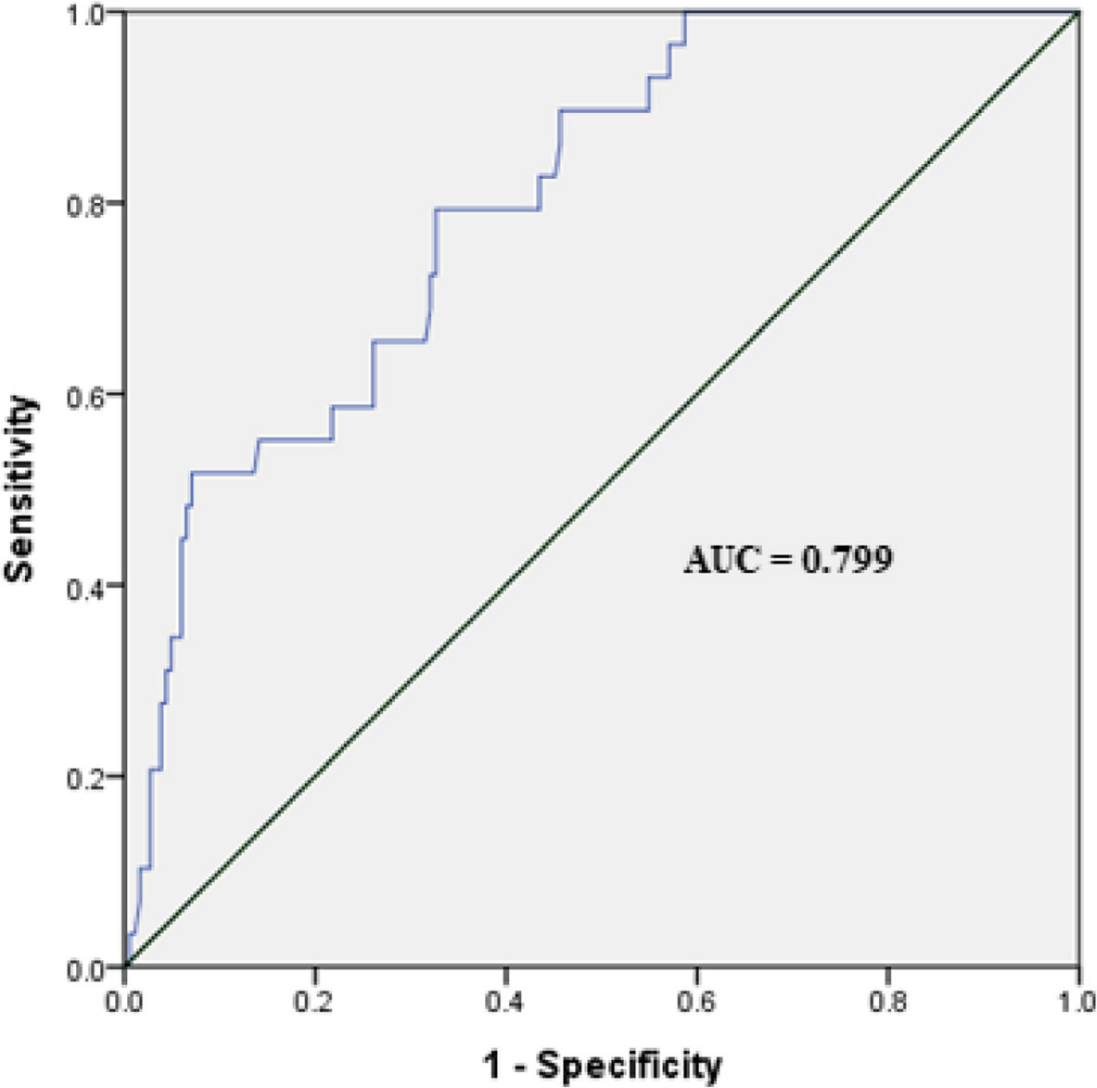
Receiver operator characteristic curve of PSA levels for predicting the presence of biochemical recurrence

**Fig. 4:**
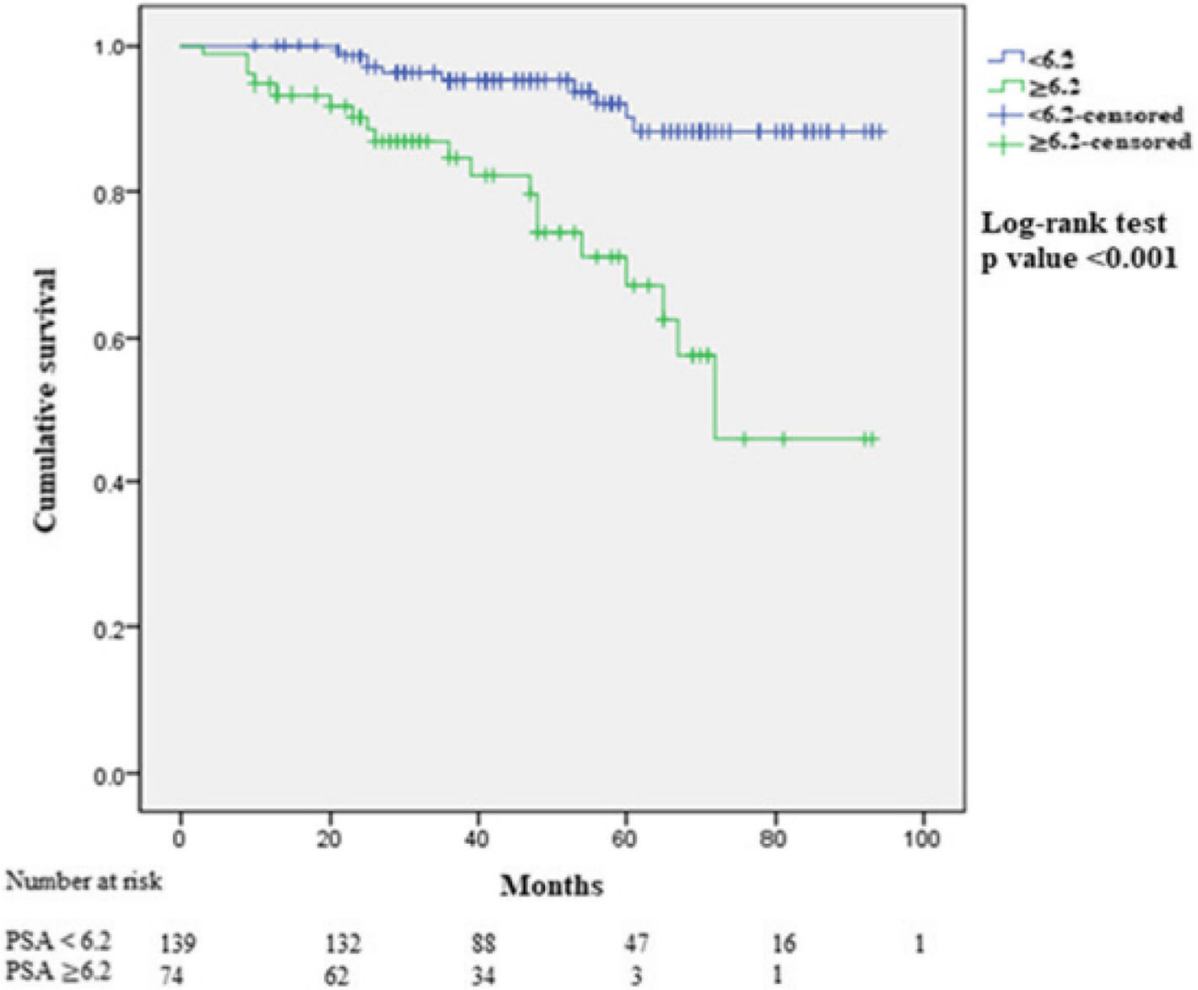
Kaplan-Meier curves for biochemical recurrence (BCR)-free survival in patients according to prostate-specific antigen levels (PSA, cut-off value 6.2)

Preoperative PSA showed a significant correlation with the presence of BCR and AP. ROC curve analysis was used to determine optimal cut-off value by the Youden Index, as shown in Fig. 2. The optimal cut-off value for PSA level that can predict BCR was determined to be 6.2 ng/ml, with an AUC of 0.799 (95% confidence interval, 0.721–0.877). This was statistically close to an excellent level. (Fig. 3) We then divided patients into two groups using the PSA cut-off value. Kaplan-Meier curves showed a significant difference between the two groups with respect to BCR (log-rank test, *p* < 0.001). (Fig. 4).

## Discussion

The widespread use of PSA testing has dramatically increased the number of low-risk PCa cases identified. Consequently, the proportion of low-risk PCa cases has increased, and treatment options for PCa are known to differ by risk classification [4]. However, several published studies have reported that the efficacy of contemporary PCa screening and diagnosis practices have limited sensitivity and specificity. The main disadvantages are failure to detect clinically significant cancer, imprecise PCa risk stratification (undersampling), and detection of small, low-risk, clinically insignificant cancers (overdetection) [21, 22]. Thus, the development of mpMRI has played a role in complementing these deficiencies.

In the past, widespread acceptance of mpMRI suffered from a lack of standardized diagnostic criteria for reporting results, leading to a substantial variability in interpretation [23]. To standardize the evaluation and reporting of prostate MRI, the European Society of Urogenital Radiology published guidelines based on an expert consensus in 2012, termed the Prostate Imaging Reporting and Data System (PI-RADS). This system was then upgraded to PI-RADS version 2 in 2015. As such, the role of mpMRI in diagnosing PCa is growing [15, 16].

The standardization of prostate mpMRI and the subsequent development of platforms for MRI-targeted biopsy represent a potential tool to the overcome limitations of conventional TRUS-biopsy. In addition, this has had a significant impact on the treatment direction. Notably, iPCa on mpMRI has been observed in several published studies, which is considered to be one standard of active surveillance [24–26].

In addition, some studies have identified mpMRI as an important prerequisite for prostate biopsy. Wysock et al. [27] reported that 75 patients had a pre-biopsy MRI showing no suggestion of cancer. In 74 of those patients, no cancer with GS ≥7 was found by biopsy, which translates into a remarkable 98.7% negative predictive value for potentially aggressive disease. Thus, they suggested that a negative MRI result obviates the need for a biopsy.

However, negative findings on MRI have already been reported in several studies, and MRI-iPCa is also not uncommon in the PI-RADS era. Filson et al. reported that in a consecutive series of 1042 men undergoing template biopsy, regardless of MRI findings, the incidence of clinically significant PCa in men with no MRI-suspicious lesions was 16%. [12].

Further, in a study by Le et al., when looking carefully at whole-mount prostatectomy specimens, the incidence of clinically significant PCa not seen on expert-read MRI was 28%. [8], which was the same as in our study. A significant PCa was observed in some of the pathologic specimens of 213 patients with iPCa, and then we investigated their long-term oncologic outcomes. Most cases of iPCa showed low risk for G6 (76.1%), but PCa of G8 or higher was observed in 20 cases (6.6%), and T3 pathology was observed in 24 cases (13.1%). BCR was observed in 29 cases (13.6%) during the follow-up period. This showed that there may be AP and oncologic outcomes among invisible cancers.

In addition, 55 patients with iPCa who had a low risk of G6 in biopsy showed an upgraded feature of G7 or above in the final pathology. The possibility of upgrading to G6 has already been reported in many articles [19, 28]. We agree that some GS 6 cases thought to be insignificant may have the biological potential for de-differentiation. This suggests that in patients with a low risk of G6, iPCa on mpMRI finding is not a prerequisite for AS. Our findings will be able to complement this. We found that PSA is closely related to oncologic outcome and AP. Through this, we determined the optimal PSA cut-off value to predict BCR during the follow-up period. PSA > 6.2 ng/ml could be considered as a basis for determining the active treatment of iPCa. PSA is still the most reliable marker of PCa in the era of mpMRI and PI-RADS.

However, our study had several limitations. First, this was a retrospective review of data from patients treated at a single institution; therefore, multi-center, prospective studies are still needed. Second, as we previously mentioned, mpMRI scans were obtained after prostate biopsy. There may be some limitations on image interpretation, such as hemorrhages, compared to pre-biopsy images [29]. However, Park et al. reported that no consensus has yet been reached regarding the optimal timing of MRI for acute staging [30], although pre-contrast T1W images were always included in MRI protocols to improve image quality. This was because areas of hemorrhage appeared characteristically hyper-intense on T1W, which reduced the impact of hemorrhage in the interpretation of these images. Additionally, only patients with MRI images where there was no image interpretation restriction, based on PI-RADS grade determined by the experienced uroradiologist, were selected. Patients with any limitations of interpretation owing to pre-biopsy images were excluded. Despite these limitations, our study remains informative for clinicians who treat patients with iPCa on mpMRI.

As for the strengths of our study, to the best of our knowledge, this is the first investigation of long-term oncologic outcomes and prognostic factors in patients with iPCa based on PI-RADS who underwent RP. Furthermore, we investigated the long-term follow up oncologic outcomes of iPCa with an established protocol. No patients were treated with adjuvant androgen deprivation therapy or radiotherapy until BCR. This allowed us to observe the natural history of BCR after RP. Based on this, our study provides criteria for predicting adverse oncologic outcomes of patients with iPCa without further preoperative examination. Clinical information, especially increased PSA, may help to determine the direction of treatment for patients with iPCa beyond the use of MRI data alone. This may be a criterion for clinicians to consider a systematic prostate biopsy for iPCa or to administer aggressive treatment in patients with PCa that has already been diagnosed.

Our study used mpMRI as a preoperative image modality. Recently, modalities, such as prostate-specific membrane antigen ligand positron emission tomography, have been used for the diagnosis and treatment of prostate cancer, recently. We believe that the development of these modalities may be a way to overcome mpMRI limitations observed in our study [31, 32].

## Conclusions

Our results demonstrate that iPCa on mpMRI can have AP, and its oncologic prognosis is not always good. Therefore, invisible tumors on mpMRI do not appear to predict insignificant clinical implications. Among such tumors, there is a clinically significant cancer incidence. In addition, our results showed that PSA is the most clinically reliable predictor of pathologic outcome and prognosis of iPCa. Specifically, PSA above 6.2 ng/ml was significant in iPCa in relation to its oncologic prognosis. In the present results, we suggest that aggressive treatment such as RP should be performed in patients with invisible cancer on mpMRI if it is clinically necessary, such as in patients with elevated PSA > 6.2 ng/ml. Finally, when prostate biopsy is clinically needed, such as in cases with PSA elevation, a negative mpMRI finding should not be a prerequisite for excluding systematic biopsy.

## Abbreviations

ADC: Apparent-diffusion coefficient
ADT: Androgen deprivation therapy
AP: Adverse pathology
AUC: Area under the curve
BCR: Biochemical recurrence
BMI: Body mass index
CI: Confidence interval
DCE: Dynamic contrast enhanced imaging
DM: Diabetes mellitus
DWI: Diffusion-weighted imaging
ECE: Extracapsular extension
GS: Gleason score
HR: Hazard ratio
iPCa: Invisible prostate cancer
IQR: Interquartile range
LVI: Lymphovascular invasion
mpMRI: Multiparametric magnetic resonance imaging
MRI: Magnetic resonance imaging
OR: Odds ratio
PCa: Prostate cancer
PI-RADS: Prostate Imaging Reporting and Data System
PNI: Perineural invasion
PSA: Prostate-specific antigen
PSM: Positive surgical margins
ROC: Receiver operator characteristic
RP: Radical prostatectomy
RT: Radiation therapy
SVI: Seminal vesicle invasion
T1W: T1-weighted
T2W: T2-weighted
TRUS: Transrectal ultrasound
